# The unique evolution of the pig LRC, a single KIR but expansion of LILR and a novel Ig receptor family

**DOI:** 10.1007/s00251-018-1067-1

**Published:** 2018-06-21

**Authors:** John C. Schwartz, John A. Hammond

**Affiliations:** The Pirbright Institute, Pirbright, Surrey, GU24 0NF UK

**Keywords:** Annotation, Immunoglobulin domains, Leukocytes, Natural killer cells, Pseudogenes, *Sus scrofa*

## Abstract

The leukocyte receptor complex (LRC) encodes numerous immunoglobulin (Ig)-like receptors involved in innate immunity. These include the killer-cell Ig-like receptors (KIR) and the leukocyte Ig-like receptors (LILR) which can be polymorphic and vary greatly in number between species. Using the recent long-read genome assembly, Sscrofa11.1, we have characterized the porcine LRC on chromosome 6. We identified a ~ 197-kb region containing numerous *LILR* genes that were missing in previous assemblies. Out of 17 such *LILR* genes and fragments, six encode functional proteins, of which three are inhibitory and three are activating, while the majority of pseudogenes had the potential to encode activating receptors. Elsewhere in the LRC, between *FCAR* and *GP6*, we identified a novel gene that encodes two Ig-like domains and a long inhibitory intracellular tail. Comparison with two other porcine assemblies revealed a second, nearly identical, non-functional gene encoding a short intracellular tail with ambiguous function. These novel genes were found in a diverse range of mammalian species, including a pseudogene in humans, and typically consist of a single long-tailed receptor and a variable number of short-tailed receptors. Using porcine transcriptome data, both the novel inhibitory gene and the LILR were highly expressed in peripheral blood, while the single *KIR* gene, *KIR2DL1*, was either very poorly expressed or not at all. These observations are a prerequisite for improved understanding of immune cell functions in the pig and other species.

**Electronic supplementary material:**

The online version of this article (10.1007/s00251-018-1067-1) contains supplementary material, which is available to authorized users.

## Introduction

The leukocyte receptor complex (LRC) encodes a diverse array of immunoglobulin (Ig)-like genes crucially involved in innate and cell-mediated immune responses against intracellular pathogens and neoplasms. The LRC is highly variable in gene content across mammals, including between closely related species. Two known subgroups of LRC-encoded receptors are variably expanded in mammals: the killer-cell Ig-like receptors (KIR) and the leukocyte Ig-like receptors (LILR). The KIR and the functionally equivalent, yet unrelated, killer-cell lectin-like receptors (KLR) mediate natural killer (NK) cell functions through their interactions with polymorphic major histocompatibility complex (MHC) class I or class I-like ligands. The Simiiformes (i.e., simians) and the Bovidae (e.g., cattle, sheep, and goats) are the only known clades that have expanded their complement of *KIR* genes (Guethlein et al. 2007; McQueen et al. 2002; Sanderson et al. 2014; Storset et al. 2003), which are also highly polymorphic and variable in gene content between haplotypes. In contrast, mice (*Mus musculus*) have a highly expanded and polymorphic repertoire of *KLRA* genes, but possess only two *KIR*-like genes outside the LRC and which are thought to have alternative functions (Bryceson et al. 2005; Hoelsbrekken et al. 2003). Humans possess 11 functional *LILR* genes that are expressed by a wide variety of lymphoid and myeloid cell types, including NK cells, and two *LILR* pseudogenes. Although the ligands for many of them are unknown, at least five *LILR* are known to interact with MHC class I ligands (Zhang et al. 2017). In mice, the paired Ig-like receptors (PIR) are orthologous to human LILR, and some of these also interact with MHC class I ligands (Kelley et al. 2005; Liang et al. 2002; Takai 2005).

Outside of primates and rodents, *LILR* orthologs are variably and independently expanded, and the cell types that express them and their ligand specificities remain unknown. Cattle (*Bos taurus*), for example, have a highly expanded repertoire of approximately 26 *LILR* genes (Hogan et al. 2012), while the goat (*Capra hircus*) has nine (Bickhart et al. 2017). Pigs (*Sus scrofa*) appear to possess a very limited repertoire of potential NK cell receptors. These include a minimal set of *KLR* genes in the NK complex on chromosome 5 (Schwartz et al. 2017), a single *KIR2DL1*, and three described *LILR* genes in the LRC of questionable function based on sequence alone (Guethlein et al. 2015; Sambrook et al. 2006). However, the previous characterization of the porcine LRC utilized BAC clones that only covered 273 kb and did not extend beyond the third intact *LILR* gene. The porcine reference assembly, Sscrofa10.2, was later used to identify immune genes across the genome, but this analysis also found a lack of potential NK cell receptors (Dawson et al. 2013). Although species with limited *KIR* and *KLR* diversity have been described before, including in species with diverse MHC class I gene content (Hammond et al. 2009, 2012), without a complete map of the LRC, it is impossible to determine the entirety of the pig NK cell receptor repertoire.

The Sscrofa10.2 assembly relied on relatively short reads (Groenen et al. 2012), which complicate the assembly of repetitive regions such as the LRC (Bickhart et al. 2017; Sanderson et al. 2014). Recently, however, the genome assembly was updated to Sscrofa11.1 using sequence from the same individual, but generated using long-read single molecule real-time sequencing technology (Pacific Biosciences). This strategy promises more accurate and complete assemblies of polymorphic and repetitive gene complexes as recently demonstrated for the goat (Bickhart et al. 2017). We therefore presently describe the LRC gene content in the latest assembly, which includes substantially expanded *LILR* gene content and a gene encoding a novel Ig-like receptor. We additionally describe the expression of these genes in porcine peripheral blood.

## Methods

### Genomic sequences

The new long-read assembly (Sscrofa11.1) for chromosome 6 was acquired from GenBank (accession no.: CM000817.5). As new assembly was generated using material from the same Duroc sow that was used for the previous assembly, Sscrofa10.2 (Groenen et al. 2012), we sought to compare the two. We therefore acquired the LRC region in Sscrofa10.2 (chromosome 6; 53,155,807–53,837,252) from the Ensembl Genome Browser (Cunningham et al. 2015). As our analyses identified novel genes in the porcine LRC, we sought additional contiguous sequences that included both the *NLRP2* and *NLRP7* genes. In the pig, this included the BAC clone PigE-173F2 (GenBank: CR853303.6) which was previously sequenced and described by Sambrook et al. (2006).

To facilitate interspecies comparisons, we also assessed gene content in the human LRC, which is *KIR* haplotype A, in the current genome assembly (GRCh38.p12; chromosome 19; 54,019,000–55,071,000). The basic local alignment search tool (BLAST) (Altschul et al. 1990) was used to identify contigs in Ensembl containing these genes in other species. These include the goat (ARS1, chromosome 18, positions 61,194,261–64,200,708), sheep (*Ovis aries*; OARv4.0, chromosome 14, positions 59,574,380–59,580,657), cattle (ARS-UCD1.2, chromosome 18, positions 62,448,480–62,454,714), the horse (*Equus caballus*; EquCab2, chromosome 10, positions 24,052,839–24,309,448), the little brown bat (*Myotis lucifugus*; Myoluc2.0, scaffold GL430447), and the Sunda flying lemur (*Galeopterus variegatus*; G_variegatus-3.0.2, Scaffold3821).

### Gene annotation and phylogenetic analysis

The genomic sequences were downloaded and manually annotated using Artemis (Rutherford et al. 2000). This process was aided by the conserved domain search tool (Marchler-Bauer and Bryant 2004) to identify putative Ig-like domains and BLAST to compare the genomic sequence to NCBI RefSeq entries and known LRC sequences in other species such as cattle (Hogan et al. 2012; Sanderson et al. 2014). Putative transmembrane (TM) regions were predicted using TMHMM (Krogh et al. 2001). Immunoreceptor tyrosine-based inhibition motifs (ITIMs) in the intracellular tail were identified based on the canonical (I/L/V/S)xYxx(L/V) motif (Orr and Lanier 2010). Genome assemblies were compared using recurrence plots generated using DOTTER with a sliding window of 200 bp (Sonnhammer and Durbin 1995). For phylogenetic analysis, nucleotide coding region sequences for the extracellular regions of the LRC Ig-like genes were aligned using MAFFT (Katoh et al. 2002), and a minimum evolution phylogenetic tree was constructed using the pairwise deletion method and maximum composite likelihood of pairwise distances (Tamura et al. 2004) and 1000 bootstrap iterations within MEGA7 (Kumar et al. 2016).

Our phylogenetic analysis identified two distinct clades of porcine *LILR* genes that we designated group 1 and 2. We also preserved the *LILRA* and *LILRB* designations used for other species to indicate whether the encoded receptors are activating or inhibitory, respectively. Thus, for example, *LILR2B10* is a group 2 inhibitory *LILR* gene and the tenth *LILR* gene identified in the present study.

### Polymerase chain reaction of KIR2DL1

As previous work identified a D1-like Ig domain fragment encoded within the *KIR2DL1* gene (Hammond et al. 2009), we investigated *KIR2DL1* mRNA splicing using PCR and cDNA from six animals leveraged from a recent MHC genotyping study (Schwartz et al. 2018). Oligonucleotide primers were designed using Primer3 (Rozen and Skaletsky 2000) and supplied by Integrated DNA Technologies, BVBA (Leuven, Belgium). Positive-sense primers were designed to amplify from either the leader region (5′-GACTCTCAGGATCATCAGCC), the D0 Ig-like domain (5′-ACAAGTCCTACCTGTCTGCC and 5′-AGTGTCACTCGCATCTCAGA), or the D2 Ig-like domain (5′-CTCCGAGAGTTCCTTCGACA). Negative-sense primers were designed to amplify from the TM region (5′-CATGGTGCCAGCAATGGATG and 5′-AAGACCCAGCCAGACCATG). Reactions were performed using DreamTaq Green PCR Master Mix (Fisher Scientific UK Ltd., Loughborough, UK) and manufacturer’s guidelines for thermal cycling conditions.

### Transcription analysis

To further determine whether or not the putative LRC genes are expressed, we leveraged a previously generated porcine transcriptome (Liu et al. 2016). Briefly, this dataset (BioProject: PRJEB12300) contains approximately 6.3 × 10^8^ paired-end 2 × 100 bp reads generated from the peripheral blood of 31 overtly healthy post-weaning Yorkshire piglets. These reads were downloaded and locally searched for sequences corresponding to the putative coding region sequence (CDS) for each porcine LRC Ig-like gene using BLAST+ (Camacho et al. 2009) and an E-value cutoff of 1 × 10^−10^. The number of sequenced fragments per kilobase of transcript per million sequenced reads (FPKM) was calculated for each gene in each sample and in the entire dataset in order to normalise expression by accounting for transcript length and number of sequenced reads.

Due to the high identity between many of the *LILR* genes, one RNA-Seq read often aligns to more than one gene. However, the two *LILR* subgroups at most share 71% identity and we did not detect individual reads mapping to both subgroups, indicating that we could accurately differentiate broad gene expression between the two clades. We also attempted to differentiate between the specific functional *LILR* genes to determine specific gene expression using the highest scoring hit from each read with ≥ 99% identity to the reference gene. As the most similar *LILR* genes are approximately 95% similar (*LILR2B10* vs. *LILR2A11*), this 99% cutoff is specific, albeit not particularly sensitive. Thus, this cutoff likely undercounts the number of reads for the specific *LILR*, especially when considering likely allelic variation. This method therefore illustrates gene expression with high confidence, but is not particularly useful for comparing expression levels between genes. The extracellular region of the novel Ig-like genes is also nearly identical to one another, so as with the *LILR*, we first identified all reads that mapped to either the long- or short-tailed form. However, as the TM and intracellular regions are unique to each of the two novel genes, we used only these high-confidence regions to differentiate their transcription.

## Results

### The organization of the porcine LRC compared to human

**Fig. 1:**
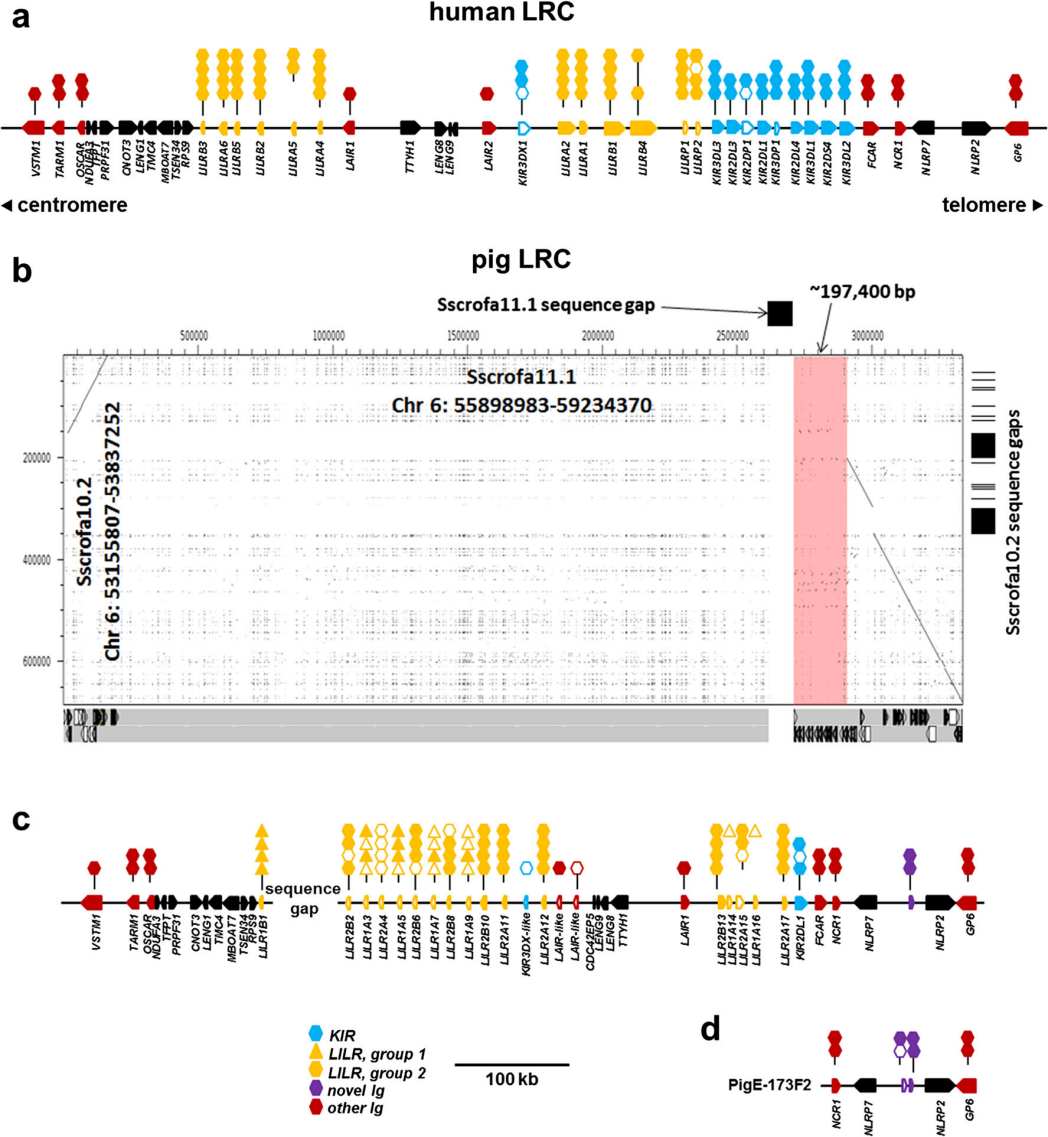
Structure and organization of the LRC in **a** humans and **b**–**d** pigs. **a** Organization of the human LRC, with *KIR* haplotype A, in the current genome assembly GRCh38.p12. **b** Recurrence plot comparing the Sscrofa10.2 (*y*-axis) and Sscrofa11.1 (*x*-axis) assemblies. Sequence gaps are indicated as black boxes above and at right. LRC-associated genes and their respective orientations in the Sscrofa11.1 assembly are indicated below the recurrence plot. Red shading indicates a region of approximately 197 kb that is present in the Sscrofa11.1 assembly, but absent from the Sscrofa10.2 assembly. **c** Organization of the LRC in Sscrofa11.1 with the contig inversion at the centromeric end corrected. The entire region spanning *VSTM1* to *GP6* is approximately 849 kb, excluding the poly-N sequence gap. **d** Organization of genes on the BAC clone PigE-173F2 (GenBank: CR853303), which was previously described by Sambrook et al. (2006) and re-annotated here. Parts **a**, **c**, and **d** are drawn to the same scale (shown at bottom) and the centromeric/telomeric orientation is the same in pigs and humans. Gene orientation is indicated with arrows pointing in the direction of transcription. Gene families are differentially colored to ease visualization. Ig-like domains are indicated as either hexagons or triangles above the respective gene, and sub-group domain phylogeny is indicated by its vertical position (e.g., human *LILRB4* is lacking both the second and third Ig domains). Open symbols indicate non-functional domains and pseudogenes, whereas closed symbols indicate that they are putatively functional. Long- or short-tailed intracellular domains are also shown to indicate whether a gene encodes an inhibitory or activating receptor, respectively

We queried the gene organization of the human LRC on chromosome 19 in the GRCh38.p12 reference assembly (Fig. 1a) in order to facilitate comparisons to the pig, which has a similarly organized LRC (Fig. 1b–d). The porcine LRC is located on the long arm of chromosome 6 (Sambrook et al. 2006), and in Sscrofa11.1 is approximately 111 Mb from the telomere. A single sequence gap exists in the middle of the LRC that separates two contigs of 2,659,904 and 4,573,057 bp (Fig. 1b). Gene organization and recurrence plot comparisons with other genome assemblies including Sscrofa10.2 indicate that the smaller contig is inverted in the assembly (Fig. 1b). The porcine LRC is typically organized as in other species; however, compared to the human LRC, the centrally positioned *LAIR1-TTYH1-LENG8-LENG9-CDC42EP5* gene cluster is inverted in the pig, with *LAIR1* and the *KIR*-proximal *LILR* being juxtaposed (Fig. 1c). There are two additional and non-functional *LAIR*-like genes proximal to *CDC42EP5* in the equivalent position as *LAIR2* in the human. Near *LAIR2* in humans is a *KIR3DX* lineage gene separated from the rest of the *KIR* region by several *LILR* (Fig. 1a). In the pig, we identified a *KIR3DX*-like D2 domain fragment in a similar position (Fig. 1c), suggestive of common ancestry.

There are 14 sequence gaps across the LRC in the short-read Sscrofa10.2 assembly, and all of these are between the *KIR*-proximal *LILR* and *VSTM1* (Fig. 1b). Comparison between assemblies revealed an additional ~ 197 kb of sequence in the new long-read assembly that had previously been obscured by one of these sequence gaps. Notably, manual annotation identified a total of 17 *LILR* genes and related fragments in the long-read assembly, including the three *LILR* that were previously reported by Sambrook et al. (2006). Thus, the sequence gaps within the previous short-read assembly obscured the vast majority of the expanded *LILR* region (Fig. 1b).

### Porcine LILR have expanded and represent two divergent lineages

**Fig. 2:**
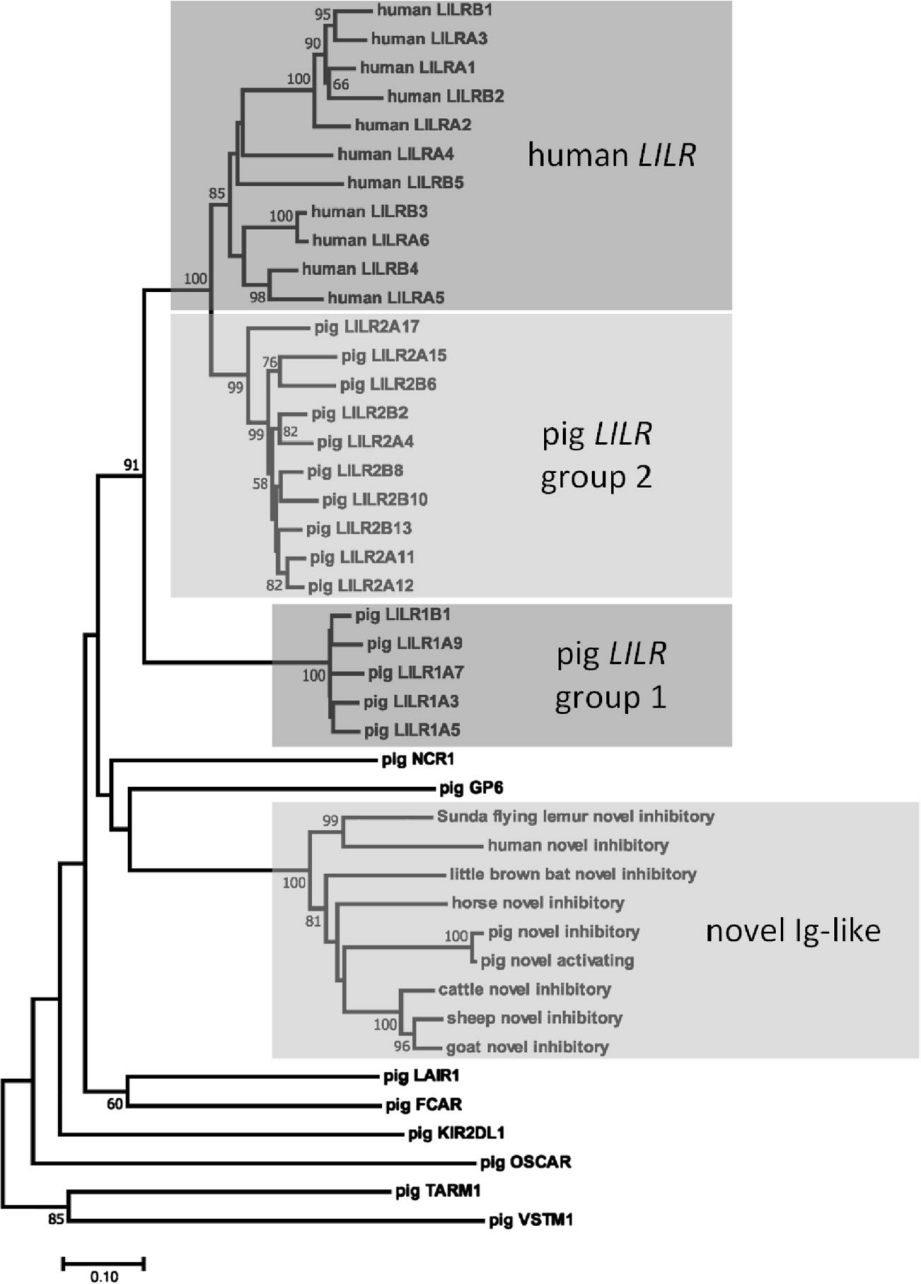
Phylogenetic analysis of Ig-like genes in the LRC using nucleotide extracellular coding region sequences. All intact porcine LRC Ig-like genes are shown to illustrate that the *LILR* and novel Ig-like genes form distinct clades. For comparison, all functional human *LILR* are shown, as well as the novel inhibitory genes from human, Sunda flying lemur, horse, little brown bat, cattle, sheep, and goat. Branch node values indicate the percentage of replicate trees in which the associated sequences clustered together (based on 1000 bootstrap replications) and only values > 50% are indicated. Branch length units are the number of substitutions per site. Except for the novel short-tailed gene sequence, which is derived from PigE-173F2, all porcine sequences are derived from the Sscrofa11.1 assembly. Genome coordinates for the non-porcine novel inhibitory genes are described in the “Methods” section. The GenBank accession numbers for the human *LILR* genes depicted here are as follows: *LILRB1* (NM_006669.6), *LILRB2* (NM_005874.4), *LILRA1* (NM_006863.3), *LILRA2* (NM_001130917.2), *LILRA3* (NM_006865.4), *LILRB3* (NM_001081450.2), *LILRB4* (NM_001278426.3), *LILRB5* (NM_001081442.2), *LILRA4* (NM_012276.4), *LILRA5* (NM_021250.3), and *LILRA6* (NM_024318.3)

Of the 17 identified *LILR* genes and fragments, only six are functional, nine are intact pseudogenes, and two are fragments of *LILR*-like Ig domains (Supplementary Table 1). Three of the functional genes are putatively inhibitory as they encode long intracellular tails with two ITIMs each, and the remaining three are putatively activating as they encode a charged arginine residue in their TM domain allowing them to interact with an activating adapter molecule. In contrast, six of the nine pseudogenes have activating potential in the TM domain and no ITIMs in the intracellular tail. Comparison of the disabling mutations between the pseudogenes indicates that they were selectively disabled following their expansion, as the mutations are different between them (Supplementary Table 1). Phylogenetic analysis of all of the Ig-like domains revealed that the porcine *LILR* are separated into two divergent groups, hereafter referred to as groups “1” and “2” (Fig. 2). Of these, the ten group 2 genes are most similar to the *LILR* genes found in humans, but have independently expanded, whereas the remaining group 1 genes are phylogenetically distinct. Peculiarly, only a single group 1 gene (hereafter provisionally named *LILR1B1*) remains functional compared to five functional group 2 genes. *LILR1B1* is also the only putatively inhibitory group 1 gene (Fig. 1b and Supplementary Table 1). The genes between the two *LILR* clades are only 63.5 to 70.5% identical such that there are few obviously shared features between them apart from their general structure and domain organization.

### The six functional LILR are transcribed in porcine peripheral blood

**Fig. 3:**
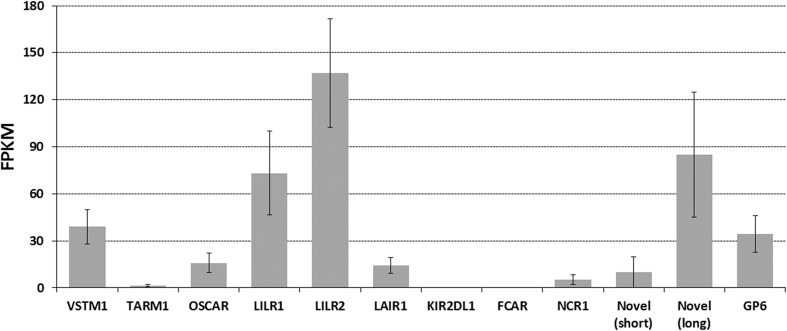
Expression of LRC Ig-like genes in porcine peripheral blood. Read numbers were normalized to fragments per kilobase of transcript per million sequenced reads (FPKM) in order to account for differences in transcript lengths and sequencing depth. Error bars indicate one standard deviation based on FPKM values for each animal. The values for *LILR* are based on reads that map to either of the two clades shown in Fig. 2. The values for the novel Ig-like genes are based on reads that map only to the region of the gene encoding the intracellular tail, as this region is unique between the two genes. All of the data, including numbers of reads, are shown in Supplementary Table 2

To confirm that the *LILR* genes are transcribed in the pig, we searched a recently published porcine transcriptomic dataset (BioProject: PRJEB12300) containing approximately 6.3 × 10^8^ paired-end reads generated from the peripheral blood of 31 post-weaning Yorkshire piglets (Liu et al. 2016). This identified a total of 207,276 reads corresponding to *LILR*, of which 72,195 were derived from group 1 (FPKM = 73.16) and 135,081 were from the group 2 *LILR* (FPKM = 136.89) (Fig. 3 and Supplementary Table 2). As the *LILR* genes within each group are highly similar to each other, multi-mapping is problematic for assessing individual gene usage. Nevertheless, using a cutoff value of ≥ 99% nucleotide identity to the reference CDS for each gene, we could account for 123,990 reads. This indicated that all of the putatively functional genes are transcribed (Supplementary Table 2).

### KIR2DL1 is very poorly expressed in porcine peripheral blood

As in previous assemblies, the single porcine *KIR* gene, *KIR2DL1*, is flanked by *NCR1* and the *LILR* genes (Fig. 1b). It has previously been suggested that *KIR2DL1* may be non-functional due to the presence of a D1-like domain fragment flanked by the other two Ig domains (Guethlein et al. 2015; Hammond et al. 2009). If not spliced from the final transcript, this D1 fragment would yield a non-functional protein as it contains multiple stop codons. In order to examine transcript splicing, we therefore attempted PCR using cDNA derived from peripheral blood leukocytes of six animals. None of the eight oligonucleotide primer combinations yielded a specific product from any of the cDNA libraries. In contrast, a single product of the expected size (4.4 kb) was obtained using the D2 positive-sense primer and both negative-sense primers when amplified using the genomic DNA derived from the same animals (not shown). Thus, primer specificity could not account for the lack of amplified transcript.

We further examined the expression of *KIR2DL1* using the same peripheral blood transcriptome dataset as used for the *LILR* and in total identified only 29 reads from 17 of the 31 animals (Supplementary Table 2) corresponding to an overall FPKM value of 0.045 (Fig. 3). Manual examination of these 29 reads revealed that 18 of them contain sequence specific to either D0 or D2 and two reads contain sequence from the intron between D0 and D2. However, none of the reads contained sequence specific for the D1 fragment or spanned the splice junction to include sequence from both functional Ig domains.

### Identification of a novel Ig-like receptor

Our annotation of Sscrofa11.1 revealed the presence of a hitherto undescribed gene encoding a two-domain Ig-like receptor flanked by *NLRP2* and its paralog, *NLRP7* (Fig. 1b, c). This novel receptor possesses a long (75 aa) intracellular tail, no charged residues in its TM domain, and two ITIM domains in the tail, indicating an inhibitory role (Supplementary Table 1). Investigation of Sscrofa10.2 revealed the presence of an additional, highly similar gene, albeit with a short (9 aa) intracellular tail. While this short-tailed gene does encode an arginine residue in the predicted TM domain, it is unusually close to the extracellular boundary, making its ability to interact with an activating adapter molecule uncertain. We also revisited the sequence of the BAC clone PigE-173F2, which was previously described by Sambrook et al. (2006), and confirmed the presence of both genes (Fig. 1c). These novel long- and short-tailed genes differ by one nucleotide and are identical in putative amino acid sequence in their membrane-distal Ig-like domain; however, the membrane-proximal domain differs by 13 nucleotides and seven amino acid residues, including a glutamine to stop codon (CAG>TAG) in the short-tailed gene, resulting in a truncated and likely non-functional protein. Both the BAC clone and the short-read assembly also contain an additional ~ 153 bp fragment that is 91% identical to the membrane-proximal Ig-like domain, indicating a history of gene expansion and contraction. Phylogenetic analysis of the Ig-like domains indicates that they share a common origin with the other genes of the LRC, yet are distinct, suggesting an ancient evolutionary divergence (Fig. 2).

To determine whether these novel genes are expressed, we searched the same pig transcriptome data as above (Liu et al. 2016) and identified 41,397 specific reads corresponding to these genes (Supplementary Table 2). Of those reads, only 847 contain sequence specific to the short-tailed TM and/or intracellular regions (FPKM = 10.11), compared to 20,424 reads that matched sequence specific for the same region of the long-tailed gene (FPKM = 84.97) (Fig. 3). Although there was substantial variation in expression between individual animals (Fig. 3), all expressed high levels of the inhibitory receptor (Supplementary Table 2). Furthermore, reads specific to the short-tailed gene were absent in 11 of the 31 animals, indicating it is likely missing in some haplotypes (Supplementary Table 2).

### The novel Ig-like receptor in other species

The possibility of these novel genes being of ancient origin led us to search available reference genomes of other mammalian species. This search identified orthologous genes flanked by *NLRP2* and *NLRP7* in several species, including cattle (seven genes), sheep (three genes), goats (three genes), horses (six genes), little brown bats (five genes), and Sunda flying lemurs (one gene). Peculiarly, within the little brown bat, these genes have evolved to encode four Ig domains with the domain structure Ig1-Ig2-Ig1-Ig2, rather than the two-domain structure observed in the other species. Despite the presence of a functional gene in the Sunda flying lemur, which is a primatomorph, no functional genes were identified in any other species within the same superorder (i.e., *Euarchontoglires*), including species within the primate, lagomorph, and rodent lineages. However, a full-length processed (i.e., retrotransposed) pseudogene is present in the human genome outside the LRC (Supplementary Table 3), despite the original gene being lost. This pseudogene clades with the putatively functional inhibitory gene found in other species (Fig. 2), is 66% identical to the full-length CDS of the pig inhibitory gene and 74% identical to that of the Sunda flying lemur. The pseudogene is also preserved in old world primates, but was not identified in more distantly related primates (Supplementary Table 3) or rodents.

## Discussion

We have presently investigated the gene content and expression of the porcine LRC using the latest genome assembly, Sscrofa11.1. This revealed a large sequence gap in previous assemblies that obscured several functional and non-functional *LILR* genes, as well as a *KIR*-like fragment and two non-functional *LAIR*-like genes. Our search of the LRC for additional Ig-like domains also revealed a novel family of genes flanked by *NLRP2* and *NLRP7*. These novel genes and the functional *LILR* genes are highly expressed in porcine peripheral blood, while *KIR2DL1* is not expressed, suggesting that it might be non-functional.

The location of the sequence gap in Sscrofa11.1 in the midst of the *LILR* region suggests that there may be additional *LILR* genes excluded from the current assembly; however, it is also quite possible that haplotypic variation in this region made assembly into a single sequence untenable, resulting in a sequence gap. Indeed, our analysis of individual *LILR* gene transcripts did not account for approximately 40.2% (83,286 reads) of all *LILR*-specific reads when we used a 99% identity cutoff. This suggests that there may be additional *LILR* genes or alleles that are not accounted for in the present assembly or that the pseudogenes are highly transcribed. Additional investigation is necessary to determine the extent of *LILR* polymorphism and if different haplotypes vary in gene content.

Seven of the nine intact *LILR* pseudogenes possess activating tails, and all group 1 pseudogenes are activating. This large proportion of activating pseudogenes suggests a tendency to lose their function, perhaps as a means of avoiding unnecessary stimulation and cytotoxicity. This observation is consistent with cattle *KIR* (Sanderson et al. 2014), human *KIR* and mouse *KLRA* (Abi-Rached and Parham 2005), and *KLRC* in bovids and horses (Schwartz et al. 2017). However, this phenomenon is unlike the human *LILR*, which have 11 functional genes, but only two pseudogenes (López-Álvarez et al. 2014). Furthermore, our phylogenetic analysis indicates that the porcine *LILR* have independently expanded two distinct clades compared to humans. Given that the human *LILR* are expressed on diverse cell types and recognize a diverse range of ligands, it is therefore impossible to predict the function of the porcine *LILR* or cellular expression. As such, further work is necessary to fill these gaps in knowledge.

It was previously noted that a D1-like Ig domain fragment might disrupt *KIR2DL1* and prevent its translation into a full-length protein (Guethlein et al. 2015; Hammond et al. 2009). While this alone would not necessarily prevent transcription, we found that *KIR2DL1* is barely expressed, if at all, in the peripheral blood transcriptome where one would expect it to be transcribed in NK cells. Furthermore, PCR analysis of cDNA derived from peripheral blood leukocytes also failed to detect transcribed *KIR2DL1*. These observations are in agreement with a previous microarray expression study that revealed a lack of transcription in 62 tissue/cell types using a probe specific for *KIR2DL1* (probe ID: PigE-108A11.2;001005_st) (Freeman et al. 2012). However, the animals in the present study and the previous microarray study were not immune stimulated, so *KIR2DL1* transcription may occur under different conditions than those studied.

We identified a novel Ig-like gene family in a diverse range of mammals for which genomic sequence is available. The presence of the gene family across the *Laurasiatheria* (e.g., pigs, cattle, goats, horses, bats), a functional gene in the Sunda flying lemur, and a pseudogene in primates indicate that this group of genes must have originated before the divergence of the *Laurasiatheria* and *Euarchontoglires* approximately > 92 Mya (Meredith et al. 2011). Except for the primate pseudogene, which is retrotransposed to a location outside of the LRC, the novel Ig-like genes always appear localized between *NLRP2* and *NLRP7*. We did not identify a copy of the novel gene outside of these two mammalian clades. However, given the fragmented state of most genome assemblies, poor sequence assembly and sequence gaps may obscure the gene in more distant species. Therefore, the apparent lack of the novel gene outside of the *Laurasiatheria* and *Euarchontoglires* does not preclude the possibility of its existence.

Although expressed by peripheral blood cells, the function of the novel Ig-like receptors is unknown. All of the species possessing at least one gene copy encode a single long-tailed inhibitory gene, suggesting a conserved function. In contrast, variable numbers of short-tailed receptors that possess extracellular domains that are nearly identical to those of the inhibitory gene are present across mammalian species. Furthermore, there is also likely haplotypic variation in short-tailed gene number, as evidenced by the absence of short-tailed transcripts in some pigs, and the lack of a short-tailed gene in the Sscrofa11.1 assembly. This is consistent with the observation that inhibitory receptors are likely ancestral and that paired activating receptors are rapidly gained and lost due to changing selective pressures (Abi-Rached and Parham 2005). However, the lack of obvious activating potential for the novel short-tailed receptor is vexing. This receptor pairing is perhaps reminiscent of the paired LAIR receptors encoded in the human LRC, in which the function of inhibitory LAIR1 is antagonized by soluble LAIR2 through its competition for ligand (i.e., collagen) (Lebbink et al. 2008). In this example, highly expressed LAIR2 can out-compete LAIR1 for binding sites thereby resulting in a more activated phenotype, as evidenced by the correlation of autoimmune thyroiditis with elevated expression of LAIR2 (Simone et al. 2013). Further study is therefore necessary to determine what cell types specifically express these novel receptors and to identify their ligands.

## Electronic supplementary material

ESM 1 (PDF 630 kb)
