# Supplementary material for: The unique evolution of the pig LRC, a single KIR but expansion of LILR and a novel Ig receptor family
Source: Immunogenetics. 2018 Jun 21;70(10):661–9. doi: 10.1007/s00251-018-1067-1 (PMC6182393; doi:10.1007/s00251-018-1067-1)
Supplement: Supplementary file 1 — (PDF 630 kb) [file 251_2018_1067_MOESM1_ESM.pdf]

Supplementary Material

The unique evolution of the pig LRC, a single *KIR* but expansion of *LILR* and a novel Ig receptor family

John C. Schwartz[1] and John A. Hammond[1†]

[1] The Pirbright Institute, Pirbright, Surrey GU24 0NF, UK

[†] Corresponding author

Phone: (+44)1483231397

E-mail: john.hammond@pirbright.ac.uk

Supplementary Table 1 - Genomic features of porcine *LILR*, *KIR*, and novel Ig-like genes

assembly	accession #	position of 1st base in CDS	orientation	name	functional?	length (bp)																							TM region	ITIM 1	ITIM 2	notes
						leader				immunoglobulin-like domains							stalk and TM				intracellular tail											
						exon	intron	exon	intron	exon	intron	exon	intron	exon	intron	exon	intron	exon	intron	exon	intron	exon	intron	exon	intron	exon	intron	exon				
Sscrofa11.1, chr6	NC_010448.4	55914685	+	*LILR1B1*	yes	**49**	206	**36**	172	**285**	124	**306**	221	**282**	271	**300**	541	**42**	936	**129**	342	**43**	85	**38**	195	**53**	629	**177**	PWYLSLLIGVSVAFVLLLLLLLFLL	VTYAQL	SVYATL	
Sscrofa11.1, chr6	NC_010448.4	58616348	-	*LILR2B2*	no	**34**	157	**36**	175	**285**	143	**303**	211	**295**	248	**292**	-	**-**	1456	**122**	-	**-**	-	**-**	-	**-**	-	**-**	PWYLSLLIGVLVAFILLLLLLLFLL	–	–	multiple frameshifts and stop codons, TM domain is 3701 bp from end of contig, suggesting rest of tail may be missing in sequence gap
Sscrofa11.1, chr6	NC_010448.4	58632087	-	*LILR1A3*	no	**49**	191	**36**	171	**282**	151	**306**	220	**282**	270	**304**	-	**-**	1023	**122**	-	**-**	-	**-**	-	**-**	-	**-**	TVGNLVRMATAALILLSLGILLFQA	–	–	frameshifts in 2nd and 4th Ig domains
Sscrofa11.1, chr6	NC_010448.4	58647503	-	*LILR2A4*	no	**34**	156	**36**	183	**278**	144	**303**	210	**296**	248	**302**	-	**-**	1013	**122**	-	**-**	-	**-**	-	**-**	-	**-**	TVGNLVRMATAALILLGLGILLFQA	–	–	multiple frameshifts and stop codons
Sscrofa11.1, chr6	NC_010448.4	58666026	-	*LILR1A5*	no	**49**	204	**36**	172	**282**	151	**306**	221	**281**	260	**299**	-	**-**	1019	**122**	-	**-**	-	**-**	-	**-**	-	**-**	TVDNLVRMATAALILLSLGVLLFQA	–	–	multiple frameshifts and stop codons
Sscrofa11.1, chr6	NC_010448.4	58680022	-	*LILR2B6*	no	**34**	156	**36**	184	**285**	142	**303**	213	**289**	255	**293**	-	**-**	-	**-**	-	**-**	-	**-**	537	**51**	949	**147**	–	VTYAQL	SVYAVL	multiple frameshifts and stop codons, TM region deleted
Sscrofa11.1, chr6	NC_010448.4	58699452	-	*LILR1A7*	no	**49**	209	**36**	171	**284**	152	**304**	225	**282**	270	**304**	-	**-**	1030	**170**	-	**-**	-	**-**	-	**-**	-	**-**	TVGNLVRMATAALILLSLGILLFQA	–	–	multiple frameshifts and stop codons
Sscrofa11.1, chr6	NC_010448.4	58715056	-	*LILR2B8*	no	**34**	157	**36**	183	**278**	144	**303**	213	**297**	268	**285**	-	**-**	1564	**123**	-	**-**	-	**-**	689	**51**	987	**146**	PWYLSLLIGVLVAFILLLLLLLFLL	VTYAQL	SVYAAL	multiple frameshifts and stop codons
Sscrofa11.1, chr6	NC_010448.4	58733675	-	*LILR1A9*	no	**49**	210	**36**	159	**284**	153	**306**	220	**282**	272	**300**	-	**-**	1031	**123**	-	**-**	-	**-**	-	**-**	-	**-**	TVGNIIRMAMAALILLGLGILLFQA	–	–	multiple frameshifts and stop codons
Sscrofa11.1, chr6	NC_010448.4	58743671	-	*LILR2B10*	yes	**34**	155	**36**	173	**285**	134	**303**	212	**297**	246	**303**	-	**-**	2258	**120**	-	**-**	-	**-**	689	**53**	982	**147**	PWYLSLLIGVLVAFVLLLLLLLLRH	VTYAQL	SVYAVL	
Sscrofa11.1, chr6	NC_010448.4	58768988	-	*LILR2A11*	yes	**34**	157	**36**	176	**285**	144	**303**	167	**297**	245	**303**	-	**-**	1071	**122**	-	**-**	-	**-**	-	**-**	-	**-**	TVGNIVRMAVAALILLGLGILLFEV	–	–	
Sscrofa11.1, chr6	NC_010448.4	58808088	-	*LILR2A12*	yes	**34**	157	**36**	184	**285**	145	**303**	214	**297**	248	**303**	-	**-**	1070	**122**	-	**-**	-	**-**	-	**-**	-	**-**	TVENLVRMAVAALILLGLGILLFQA	–	–	
Sscrofa11.1, chr6	NC_010448.4	58979010	+	*LILR2B13*	yes	**34**	157	**36**	183	**285**	144	**303**	214	**297**	250	**303**	-	**-**	1578	**123**	341	**43**	77	**38**	193	**53**	981	**147**	WYLSLLIGVSVAFVLLLLLLFLLRH	VTYAQL	SVYAAL	
Sscrofa11.1, chr6	NC_010448.4	58988542	+	*LILR1A14*	no	-	-	-	-	**252**	-	-	-	-	-	-	-	-	-	-	-	**-**	-	**-**	-	**-**	-	**-**	–	–	–	only 1st Ig domain which contains deletion and frameshift and stop codons, rest of gene is deleted
Sscrofa11.1, chr6	NC_010448.4	58997423	+	*LILR2A15*	no	**34**	167	**36**	184	**285**	144	**303**	214	**305**	-	**-**	1316	**51**	152	**176**	-	**-**	-	**-**	-	**-**	-	**-**	TVENLVRMAVAGLILLGLGILLFQA	–	–	single indel resulting in frameshift in 3rd Ig domain
Sscrofa11.1, chr6	NC_010448.4	59014513	+	*LILR1A16*	no	-	-	-	-	**271**	-	-	-	-	-	**-**	-	-	-	-	-	**-**	-	**-**	-	**-**	-	**-**	–	–	–	only 1st Ig domain which contains deletion and frameshift and stop codons, rest of gene is deleted, incorrect splice site
Sscrofa11.1, chr6	NC_010448.4	59043010	+	*LILR2A17*	yes	**34**	169	**36**	174	**285**	144	**303**	215	**297**	239	**303**	514	**51**	149	**122**	-	**-**	-	**-**	-	**-**	-	**-**	TVGNLVRMALAALILLGLGILLFQA	–	–	
Sscrofa11.1, chr6	NC_010448.4	59056840	+	*KIR2DL1*	no?	**34**	239	**36**	789	**285**	1305	**176**	2046	**300**	-	**-**	571	**51**	3691	**105**	-	**-**	-	**-**	450	**53**	95	**147**	LHGLAGSSGAIICFVILLFILIH	VTYTEV	SVYMDL	in-frame if 2nd Ig domain spliced out, does not appear to be expressed
PigE-173F2	CR853303.6	71439	-	*Novel-2DS1*	no	**34**	99	**36**	181	**264**	247	**300**	-	-	-	-	-	**-**	693	**134**	-	**-**	-	**-**	-	**-**	-	**-**	VRLSLAGLVLLILVGLSAEAWCS	–	–	in-frame stop codon in 2nd Ig domain, stalk region deleted
PigE-173F2	CR853303.6	67224	-	*Novel-1DP*	no	**-**	-	**-**	-	**-**	-	**157**	-	-	-	-	-	**-**	-	**-**	-	**-**	-	**-**	-	**-**	-	**-**	–	–	–	only the last half of 2nd Ig domain remains, the rest of the gene is deleted
PigE-173F2	CR853303.6	66480	-	*Novel-2DL1*	yes	**34**	99	**36**	181	**264**	247	**300**	-	-	-	**-**	221	**42**	2060	**126**	-	**-**	-	**-**	1574	**53**	76	**159**	LGLLIGAPVASGLLLLVLLLLVCCC	VTYCQL	SEYATL	
Sscrofa11.1, chr6	NC_010448.4	59167942	+	*Novel-2DL1*	yes	**34**	99	**36**	181	**264**	248	**300**	-	-	-	**-**	221	**42**	2059	**126**	-	**-**	-	**-**	1574	**53**	75	**159**	LGLLIGAPVASGLLLLVLLLLVCCC	VTYCQL	SEYATL	

Supplementary Table 2 - Expression of porcine LRC Ig-like genes in peripheral blood

Sample Accession #	total reads	*VSTM1* reads	*VSTM1* FPKM	*TARM1* reads	*TARM1* FPKM	*OSCAR* reads	*OSCAR* FPKM	*LILR* – *LILR* Group 1 Reads	*LILR* – *LILR* Group 1 FPKM	*LILR* – *LILR* Group 2 Reads	*LILR* – *LILR* Group 2 FPKM	*LILR* – >99% identity (functional genes) – *LILR1B1* reads	*LILR1B1* FPKM	*LILR2B10* reads	*LILR2B10* FPKM	*LILR2A11* reads	*LILR2A11* FPKM
ERR1199492	7164191	189	43.4616247	7	1.23368903	42	7.08030225	738	66.0335442	1136	101.6451	382	30.64411	106	9.376305	70	7.080302
ERR1199493	26793115	556	34.1871496	20	0.94250054	302	13.612986	3104	74.2632422	4556	109.0024	1153	24.73186	459	10.85632	480	12.98192
ERR1199494	20355918	507	41.0325564	17	1.05446713	374	22.1896562	1877	59.1083698	3757	118.3112	716	20.21497	376	11.7055	444	15.80568
ERR1199495	22784717	419	30.2957489	24	1.32997177	436	23.1106688	2053	57.7591392	4379	123.1989	810	20.43112	397	11.0418	419	13.32574
ERR1199496	18966867	502	43.6033069	14	0.93198142	227	14.4543951	2328	78.6797151	4766	161.0771	962	29.14944	423	14.13311	382	14.59448
ERR1199497	7989712	127	26.1868889	5	0.79015756	48	7.25570765	741	59.4514546	1353	108.5531	294	21.14789	122	9.676576	112	10.15799
ERR1199498	23554800	591	41.3351358	6	0.32162267	295	15.1255877	2511	68.3349205	6180	168.1839	986	24.05738	504	13.55951	555	17.07397
ERR1199499	13709321	122	14.6607164	0	0	91	8.01669037	488	22.8180894	1372	64.1525	241	10.10303	138	6.379052	123	6.501448
ERR1199500	9268319	196	34.8390583	1	0.13623034	163	21.2400872	1400	96.828335	2079	143.7901	586	36.33686	192	13.12784	214	16.73146
ERR1199501	19314255	341	29.0862496	11	0.71910042	219	13.6941732	2247	74.5762451	3476	115.3658	954	28.38711	354	11.61498	287	10.76775
ERR1199502	23890161	700	48.271442	25	1.32128271	541	27.3494031	2303	61.7945627	4707	126.2992	855	20.56827	452	11.98981	377	11.43518
ERR1199503	21284480	491	38.0040391	22	1.30507195	475	26.9525728	2502	75.3528465	4385	132.0632	1340	36.18199	428	12.74306	331	11.26901
ERR1199504	20612443	366	29.2524959	13	0.79632198	363	21.2689877	2630	81.7902776	3697	114.9729	1237	34.48982	382	11.74429	316	11.10909
ERR1199505	17314790	346	32.9207847	7	0.51045285	91	6.34737018	1768	65.4546393	3473	128.5769	802	26.61999	318	11.63866	310	12.97375
ERR1199506	17319993	509	48.4151609	16	1.16639887	175	12.2028142	1247	46.1523844	3253	120.3959	557	18.48239	294	10.75704	344	14.39235
ERR1199507	30251343	807	43.9481081	56	2.33732006	649	25.9101365	7660	162.31532	10332	218.935	3797	72.13511	927	19.41905	629	15.067
ERR1199508	47378520	1559	54.2095664	28	0.74619332	408	10.4003591	6410	86.7265242	11316	153.1041	3262	39.56883	1131	15.12774	875	13.38282
ERR1199509	19645971	332	27.8404271	13	0.83549657	210	12.9096795	1814	59.1887524	4258	138.9337	821	24.01709	425	13.70908	382	14.08999
ERR1199510	18029309	422	38.5606795	15	1.05047808	275	18.4214272	835	29.6881267	3167	112.6016	457	14.5676	262	9.209058	305	12.25862
ERR1199511	21649663	416	31.655815	29	1.691304	267	14.8946322	2271	67.2421197	4837	143.2189	1076	28.56353	487	14.25512	409	13.68967
ERR1199512	12472913	206	27.2088782	11	1.11352407	168	16.2671343	1400	71.9507863	2624	134.8563	630	29.02842	240	12.19372	265	15.39568
ERR1199513	17043347	429	41.4680597	13	0.96308204	183	12.9677869	3077	115.730549	4699	176.7364	1396	47.07402	469	17.43857	415	17.64469
ERR1199514	20492166	601	48.3167724	18	1.10907128	201	11.8461671	2893	90.4973725	5110	159.8485	1131	31.71944	542	16.76117	445	15.73595
ERR1199515	19791642	387	32.2136879	16	1.02073492	151	9.21435168	2228	72.1620333	3640	117.8949	881	25.58261	365	11.68703	266	9.739143
ERR1199516	20746549	527	41.8481302	32	1.94750657	249	14.4951644	2899	89.5731301	4135	127.763	1349	37.36946	377	11.51565	298	10.40858
ERR1199517	22762430	610	44.1491677	55	3.05083616	324	17.1907985	3074	86.5686493	5152	145.0884	1392	35.14563	510	14.19857	489	15.56722
ERR1199518	36177890	665	30.2823602	39	1.3611193	223	7.44442729	2480	43.9424076	5558	98.48061	1163	18.47512	529	9.26628	512	10.25528
ERR1199519	15459495	332	35.3796954	10	0.81673189	165	12.8901599	1024	42.4600064	2471	102.4596	602	22.37958	205	8.403333	210	9.843395
ERR1199520	18614694	189	16.7269674	5	0.33914774	194	12.5868046	863	29.7187334	2148	73.96969	411	12.68927	216	7.353445	185	7.201728
ERR1199521	25550205	867	55.9031162	42	2.07553337	482	22.7835982	3385	84.9258076	8583	215.3377	1929	43.38989	916	22.71925	810	22.97267
ERR1199522	16168571	621	63.2748714	48	3.74838695	364	27.1893865	1945	77.1122489	4482	177.6952	1050	37.3223	502	19.67547	407	18.24079
**Total**	**632557790**	**14932**	**38.8892065**	**618**	**1.23356797**	**8355**	**15.9520282**	**72195**	**73.1614516**	**135081**	**136.8893**	**33222**	**30.18397**	**13048**	**13.07184**	**11666**	**13.36419**

Sample Accession #	*LILR* – >99% identity (functional genes) – *LILR2A12* reads	*LILR2A12* FPKM	*LILR2B13* reads	*LILR2B13* FPKM	*LILR2A17* reads	*LILR2A17* FPKM	*LAIR1* reads	*LAIR1* FPKM	*KIR2DL1* reads	*KIR2DL1* FPKM	*FCAR* reads	*FCAR* FPKM	*NCR1* reads	*NCR1* FPKM	*Novel Ig-like* total reads	*Novel Ig-like* short tail reads	*Novel Ig-like* short tail FPKM	*Novel Ig-like* long tail reads	*Novel Ig-like* long tail FPKM	*GP6* reads	*GP6* FPKM
ERR1199492	122	12.33996	404	33.92995	36	3.511525	58	10.751421	0	0	1	0.175576	42	6.344686	210	0	0	133	48.85409	165	21.207
ERR1199493	525	14.19898	1400	31.43937	148	3.860103	336	16.654097	5	0.184584675	5	0.234736	331	13.37004	1823	33	9.19149	887	87.11979	622	21.377
ERR1199494	552	19.65031	1448	42.8003	127	4.359869	304	19.832977	0	0	0	0	132	7.017966	1089	0	0	597	77.17916	450	20.356
ERR1199495	487	15.48839	1388	36.65345	134	4.109808	205	11.948551	1	0.043411541	4	0.220826	80	3.799919	1547	31	10.15344	743	85.81468	687	27.764
ERR1199496	546	20.86018	1542	48.91677	176	6.484514	359	25.136446	0	0	2	0.132638	58	3.309485	1985	43	16.91874	999	138.6074	418	20.293
ERR1199497	161	14.60211	474	35.69572	50	4.373199	97	16.122992	0	0	0	0	47	6.366412	245	0	0	132	43.47696	190	21.897
ERR1199498	737	22.673	2105	53.7702	174	5.162145	548	30.896278	0	0	10	0.534015	96	4.410825	2436	46	14.57383	1216	135.8534	409	15.989
ERR1199499	146	7.717166	375	16.45827	59	3.007436	104	10.074475	1	0.072149429	1	0.091752	120	9.473126	703	23	12.52008	292	56.05099	792	53.196
ERR1199500	216	16.88782	634	41.15828	79	5.956436	108	15.474898	0	0	0	0	26	3.03599	991	36	28.98656	456	129.4733	501	49.775
ERR1199501	337	12.64366	1078	33.58225	166	6.006071	138	9.4886876	0	0	3	0.195378	100	5.60338	1298	39	15.06891	637	86.79164	734	34.994
ERR1199502	544	16.50064	1777	44.75456	148	4.329154	298	16.565411	1	0.041402805	2	0.105304	96	4.348908	512	0	0	288	31.72414	593	22.856
ERR1199503	532	18.11213	1937	54.75648	185	6.07392	211	13.165108	2	0.092942809	1	0.059098	37	1.881337	826	0	0	466	57.61549	686	29.678
ERR1199504	369	12.97232	1262	36.83824	116	3.932683	155	9.9863608	1	0.047986533	0	0	35	1.837666	1254	38	13.75781	572	73.02692	791	35.336
ERR1199505	401	16.78217	1190	41.35221	84	3.390177	200	15.339733	1	0.057125711	5	0.363233	83	5.187868	557	0	0	317	48.17907	542	28.824
ERR1199506	413	17.27918	1318	45.78642	102	4.115407	197	15.105098	1	0.05710855	3	0.217875	35	2.186998	492	0	0	260	39.50409	780	41.468
ERR1199507	929	22.25317	3733	74.24758	287	6.629757	357	15.67215	7	0.228877038	7	0.291062	175	6.260679	3436	100	24.66894	1579	137.358	1052	32.021
ERR1199508	1181	18.06298	3979	50.53141	405	5.973569	474	13.286233	1	0.020876965	9	0.238943	262	5.984775	2753	52	8.190625	1430	79.42751	3342	64.952
ERR1199509	516	19.03256	1547	47.37899	156	5.548958	80	5.4078111	0	0	4	0.256106	39	2.14842	1575	42	15.95405	755	101.1323	1133	53.104
ERR1199510	425	17.08169	1283	42.81703	123	4.767453	223	16.425962	0	0	1	0.069768	39	2.341065	1275	25	10.34799	583	85.09536	670	34.219
ERR1199511	491	16.4343	1662	46.1901	158	5.099955	164	10.059994	0	0	5	0.290504	59	2.949368	1534	33	11.37517	745	90.5569	1149	48.87
ERR1199512	282	16.38333	835	40.27982	93	5.210452	93	9.9019352	0	0	0	0	111	9.63126	888	15	8.974672	438	92.41078	533	39.349
ERR1199513	533	22.66174	1657	58.49741	168	6.888343	171	13.324357	1	0.05803553	3	0.221411	38	2.412997	833	0	0	464	71.64395	428	23.124
ERR1199514	529	18.70634	1675	49.18084	161	5.490329	196	12.702033	2	0.096536372	6	0.368295	51	2.693459	670	0	0	374	48.02862	510	22.917
ERR1199515	385	14.09613	1313	39.91645	144	5.084415	151	10.132116	0	0	2	0.12711	113	6.179092	527	0	0	294	39.09146	629	29.264
ERR1199516	432	15.08894	1490	43.2125	109	3.671479	217	13.890532	0	0	2	0.12126	73	3.808071	525	0	0	288	36.53112	647	28.716
ERR1199517	605	19.26006	1705	45.06867	168	5.15764	312	18.202917	1	0.043454046	5	0.276302	99	4.707004	1405	35	11.47479	679	78.49962	837	33.859
ERR1199518	579	11.59728	1661	27.62456	160	3.09056	209	7.6719908	1	0.027340447	14	0.486763	319	9.542792	1928	32	6.600882	889	64.66584	1482	37.72
ERR1199519	313	14.67135	975	37.94707	110	4.972305	145	12.455975	1	0.063981371	2	0.16273	57	3.990319	1023	22	10.61995	539	91.7508	751	44.732
ERR1199520	233	9.070285	767	24.79182	73	2.740484	158	11.272136	1	0.0531365	1	0.067574	20	1.162792	1005	26	10.42348	475	67.15125	787	38.93
ERR1199521	915	25.95061	2512	59.15538	320	8.752174	383	19.907165	0	0	10	0.49231	242	10.25059	4360	141	41.18318	2138	220.2063	846	30.489
ERR1199522	426	19.09233	1481	55.11279	166	7.174586	126	10.349132	1	0.061175455	2	0.155593	49	3.279839	1692	45	20.76998	759	123.534	569	32.405
**Total**	**14862**	**17.02543**	**46607**	**44.33227**	**4585**	**5.065234**	**6777**	**14.227949**	**29**	**0.045346799**	**110**	**0.218739**	**3064**	**5.242236**	**41397**	**857**	**10.11057**	**20424**	**84.96831**	**23725**	**34.536**

Supplementary Table 3. Novel retrotransposed pseudogene in primates.

clade	species	assembly	location	strand	length	%ID (versus human)
Hominoidea (apes)	*Homo sapiens*	GRCh38	3:158336093-158337069	rev	977	100
	Gorilla gorilla gorilla	gorGor3.1	3:159070821-159071798	rev	978	98.77
	Pan troglodytes	CHIMP2.1.4	3:162090001-162090978	rev	978	98.67
	Pongo abelii	PPYG2	3:161539622-161540604	rev	983	94.30
	Nomascus leucogenys	Nleu1.0	GL397268:12144511-12145513	rev	1003	93.62
Cercopithecidae and Platyrrhini (old and new world monkeys)	*Papio anubis*	PapAnu2.0	2:127846647-127847412	for	770	93.12
	Macaca mulatta	Mmul_8.0.1	2:145505405-145506383	rev	983	92.57
	Chlorocebus sabaeus	ChlSab1.1	15:32473742-32474720	for	983	92.57
	Callithrix jacchus	C_jacchus3.2.1	17:19079430-19080400	for	985	89.14
Tarsiidae and Strepsirrhini (prosimians)	*Tarsius syrichta*	tarSyr1				
	Microcebus murinus	Mmur_2.0	**Not Found**			
	Otolemur garnettii	OtoGar3				
